# The effects of event-based drawing and comfort-drawing in an interview context on memory and intrusions

**DOI:** 10.1080/00049530.2026.2760024

**Published:** 2026-11-12

**Authors:** Georgina A. Maddox, Lucy C. Walter, Glen E. Bodner, Ryan P. Balzan

**Affiliations:** Psychology, Flinders University, Adelaide, Australia

**Keywords:** Drawing, autobiographical memory, posttraumatic stress, memory, intrusions

## Abstract

**Objective:**

Given the growing popularity of arts-based interventions, our study examined the effects of drawing during an interview about a distressing event on conscious event recall and intrusions.

**Method:**

We used a single-blind design where participants who had experienced a negative/stressful event were randomly allocated to draw while sharing a negative experience (*event-drawing* condition; *n* = 20), draw something unrelated to the event while sharing (*comfort-drawing* condition; *n* = 19), or only talk about the experience (*talk-only* control condition; *n* = 20).

**Results:**

Event-drawing increased the *amount* of information reported (*d* = 0.88) and *forensically relevant* statements regarding event locations (*d* = 0.73) during interviews compared to talk-only. Comfort-drawing did not have the same memory benefits as event-drawing and neither event-drawing nor comfort-drawing during an interview influenced intrusions compared to talk-only.

**Conclusions:**

Future research is needed to confirm whether the conscious recall benefits of event-drawing during a distressing experience extend to event *accuracy*. Continued research on the effects of drawing on autobiographical conscious recall and intrusions will enable updating of best practices for drawing interventions in interviews.

## KEY POINTS

**What is already known about this topic:**

1. Both event-drawing and comfort-drawing are widely used in forensic (e.g., police interviews) and non-forensic contexts (e.g., art therapy).
2. Drawing involves known memory enhancers (e.g., mental imagery, elaboration, motor skills).
3. Event-drawing can increase conscious recall of forensically relevant information (e.g., locations, objects and people) for both adults and children during interviews.

**What this topic adds:**

1. Event-drawing (compared to talking only) during an interview may increase conscious recall of forensically relevant information for adults who have shared a *distressing* experience.
2. Event-drawing is superior to other types of drawing (e.g., comfort-drawing) for increasing conscious recall of forensically relevant information.
3. It is unlikely that either event-drawing or comfort-drawing during an interview (compared to talking only) affect autobiographical memory *intrusions* following an interview.

Drawing can help individuals explore and express personal experiences that are often difficult to articulate in words alone (Maddox et al., 2024a). Indeed, verbal memory is often impaired following negative and/or stressful lived experiences (Brewin, 2014) and memories related to these types of events are largely comprised of visual-mental images (Johnsen & Asbjornsen, 2008; Strange & Takarangi, 2015). Consequently, arts-based interventions like drawing have often been used during interviews in settings such as eyewitness interviews, arts therapy and psychological assessment to facilitate communication and event recall (see C. J. Dando et al., 2020; Matsuo & Miura, 2017; see also Katz & Hamama, 2012).

Drawing involves several known memory enhancers (e.g., mental imagery, elaboration, motor skills; Wammes et al., 2019). Studies suggest that drawing can enhance memory for neutral and emotional words (Fernandes et al., 2018; Meade et al., 2019; Tran & Fernandes, 2023) and autobiographical memories during “reminiscing sessions” (Tran et al., 2023). In interview type settings (i.e., eyewitness interviews, counselling), numerous studies have found that incorporating drawing can enhance the amount of information reported about events relative to more traditional *talk-only* interviews (for review, see Driessnack, 2005; see also Maddox et al., 2024a). Yet, to date, only a few studies have examined the effects of drawing in an interview setting on autobiographical memory for personal experiences, whether trauma-related (Katz & Hamama, 2012; Katz & Hershkowitz, 2010) or emotionally laden (Gross & Hayne, 1998; Salmon et al., 2003; Wesson & Salmon, 2001). Most previous studies have focused on examining the effects of drawing on memory for neutral live/staged, or videoed events in controlled settings (e.g., C. Dando et al., 2009; Krackow, 2012; Matsuo & Miura, 2017; for review, see Maddox et al., 2024a). In fact, a recent meta-analytic review (Maddox et al., 2024a) highlighted the paucity of studies in this research area, as only 5 randomised controlled trials (RCTs) examining the effectiveness of drawing on *autobiographical* memory were identified.

## The effects of drawing on memory

In a seminal study, Gross and Hayne (1998) randomly assigned 40 children (3–4 yrs and 5–6 yrs) to either draw and talk about a time when they were happy, sad, scared, or angry, or to talk-only. Children were prompted with, “can you tell (or draw) me anything else about the time you felt happy [or sad, or scared, or angry]”. Verbal interviews with the children were conducted by the same researcher, and the emotion being elicited during questioning was counterbalanced (i.e., sad, happy, scared, angry). Because *accuracy* cannot be ascertained when using personal experiences (rather than live/staged, or videoed), the *amount of information reported* was used to gauge autobiographical memory. To score memory performance, the researchers measured how many sentences in the transcribed interviews contained a “clause” which they defined as any sentence including a verb. Each clause was classified as an *object*, *place*, *person*, *action*, or *description*. Spoken clauses not related to the experience (such as requests for more crayons) were excluded. Initial analysis indicated that the emotion being discussed did not influence the results, thus the researchers collapsed the data across emotion types. In doing so, they found that children given the opportunity to draw while talking during their interview reported twice as much information as those in the talk-only condition.

Such results were replicated by Wesson and Salmon (2001), Salmon et al. (2003) and Katz and Hershkowitz (2010), who added an additional classification capturing affective *statements* (e.g., “I felt scared”). While drawing elicited more forensically relevant information about *objects* than talk-only, no differences were found for the other five categories (place, person, action, description, affective statements). However, Katz and Hershkowitz (2010) found that children who drew while sharing their *traumatic* experiences (i.e., abuse) were more likely to provide information on specific *people*, *action*s, *locations* and *times* associated with the alleged abuse than those who did not draw. Interestingly, Wesson and Salmon also examined *interview duration*, finding that interviews with children who were asked to draw the events while talking lasted over twice as long as those only afforded the opportunity to talk (25 min vs. 10 min). Taken together, these studies suggest that the memory advantage of drawing one’s experiences during interviews is a replicable effect.

More recently, researchers have begun to examine the effects of drawing on autobiographical memory in *adults* and have reported similar findings. Indeed, a meta-analysis by Maddox et al. 2024a found that participant age (i.e., from 5–60+ years) was not a moderator of the relationship between drawing and increased event recall. Additionally, Deeb et al. (2021) found that drawing an event while talking during an interview was more effective for eliciting information from adults than only talking. Thus, the benefits of drawing on autobiographical memory recall, as well as the practical benefits (e.g., more complete, accurate recall in forensic interviews) in both child and adult populations are clear.

## Conscious recall vs intrusions

To date, studies have measured the effects of drawing on *voluntary* memory (i.e., conscious recall) but have yet to examine *involuntary* memory (i.e., intrusions) – an important type of memory that is frequently experienced following negative, stressful and/or traumatic experiences (Marks et al., 2018). Unlike consciously recalled memories that are retrieved purposefully and effortfully, intrusions are spontaneous and unintentional (Mace, 2005). Intrusions are often described as *vivid, visual, sensory* and *distressing* (Brewin, 2014; Mace, 2005) and are considered a key diagnostic criterion of posttraumatic stress disorder (PTSD; American Psychiatric Association, issuing body, & American Psychiatric Association Publishing, 2022). *Verbal cues* are known to elicit increased intrusions about traumatic experiences (Mace, 2005; Plimpton & Kvavilashvili, 2025), and as such, *talking only* in a forensic interview about a traumatic experience is likely to elicit many negative intrusions about the event being discussed. It is less clear whether the combination of a visual-motor task such as *drawing* while talking in an interview may differently affect intrusions compared to talking only.

However, recent research has found that *visual-motor* tasks such as the video game Tetris can *reduce* unwanted memory intrusions (Brewin, 2014; Holmes et al., 2009; James et al., 2015). Theoretically, visual-motor tasks are proposed to demand visuospatial resources in working memory (Brewin, 2014). These visuospatial resources are limited, and as such, engaging in visual-motor tasks after or during exposure to a negative experience (e.g., discussing a negative event during an interview) can create a competing demand for visuospatial resources (Brewin, 2014). This competing demand for resources then “interferes” with the *reconsolidation* of intrusions, reducing their frequency while leaving conscious recall intact (Brewin, 2014; James et al., 2015). However, a recent multi-lab replication study by Wessel et al. (2025) found that while the visual-motor task Tetris reduced intrusions *immediately* after exposure to a trauma analogue (i.e., trauma film), reductions did not sustain over a long-term period (i.e., over a week) compared to an active control. To the best of our knowledge, the possibility that *drawing* — also being a visual-motor task – reduces intrusions has yet to be empirically tested. Given that reducing intrusions may decrease the occurrence of other PTSD symptoms, our study aimed to address this knowledge gap, as recommended by Maddox et al. (2024a). In addition, our study aimed to examine whether the effect of drawing on intrusion reduction was dependent on the type of drawing (i.e., event vs. comfort-drawing).

Although these studies did not examine drawing or compare Tetris with a talk-only interview, they provide a theoretical basis for considering whether engaging in visuospatial processes may influence subsequent intrusive memories. However, it is important to note that differences in memory reactivation, intervention timing and the nature of the visuospatial task limit the direct application of these findings to drawing during an interview.

## Event-drawing vs comfort-drawing

Drawing has been implemented in different ways during autobiographical memory interviews. Individuals may be asked to draw their autobiographical or episodic experiences while talking (i.e., *event-drawing*, as per Gross & Hayne, 1998; Katz & Hershkowitz, 2010; Salmon et al., 2003; Wesson & Salmon, 2001), or to draw something unrelated to the experience being discussed (i.e., *comfort-drawing*; as per Poole & Dickinson, 2013; see also MacLeod et al., 2016). While event-drawing has been shown to enhance accurate event recall (for review, see Driessnack, 2005; see also Maddox et al., 2024a), little is known about the effects of comfort-drawing on memory. In fact, a recent meta-analytic review (Maddox et al., 2024a) uncovered only 2 RCTs examining the effects of comfort-drawing on memory during interviews (MacLeod et al., 2016; Poole & Dickinson, 2013).

On the one hand, MacLeod et al. (2016) suggested that comfort-drawing may deplete the cognitive resources required for complete and accurate recall. Consistent with this suggestion, they found that children directed to draw something unrelated to the target event of a memory interview made more *inaccurate* statements (i.e., reported errors) than those directed to draw the target event. MacLeod et al. concluded that best practice for the use of drawing during interviews would be to explicitly instruct interviewees to draw the event in question.

On the other hand, Poole and Dickinson (2013) suggested that *comfort-drawing* may reduce distress, a known memory inhibitor (Malta et al., 2020), which in turn may help to elicit *more* information about negative and/or stressful events. Additionally, Poole and Dickinson (2013) suggested that comfort-drawing tasks performed during interviews about distressing experiences may enhance other subjective well‑being measures, such as feelings of support. However, the researchers did not measure interview distress – a gap our current study aimed to address.

## The present study

Given the widespread use of drawing during interviews, more studies comparing the effects of event-drawing vs comfort-drawing on memory are needed. Therefore, our study sought to establish whether drawing while sharing a *distressing* experience has beneficial effects on conscious recall and/or intrusions. We measured the two memory types after randomly allocating participants to either draw their experience while verbally sharing in an interview (*event-drawing task*), draw flowers of their choosing while sharing (*comfort-drawing task*), or simply share their experience verbally (*talk-only control*). Importantly, while previous forensic studies have examined the accuracy of recalled information, the present study assesses the amount of information reported (i.e., word and statement counts) and does not provide a measure of recall accuracy.

Our study had two main aims. First, we examined whether the *amount* of information (i.e., total word count) and *type* of information (*objects*, *people*, *actions*, *locations*, *times* or *affective statements*) shared during the interviews differed as a function of the drawing task *type* (event vs comfort), relative to a talk-only control. Second, we examined whether the *frequency* of intrusions and *quality* of intrusions (i.e., vividness, valence, distress) differed as a function of the drawing task type, relative to a talk-only control.

Of the studies we reviewed herein, *all* reported some conscious recall benefit following drawing a target event during forensic style interviews. Thus, we predicted that participants afforded the opportunity to draw their experience during the interview (event-drawing condition) would report more information (i.e., greater word count) than those who talked-only. In addition, we predicted that the types of information reported (e.g., objects, people, actions, locations) during our event-drawing interviews would be greater than during our talk-only interviews, replicating the effects obtained in previous studies (see Maddox et al., 2024a for review).

Of the seven studies reviewed, only 2 examined comfort-drawing and failed to see a memory benefit (see MacLeod et al., 2016; Poole & Dickinson, 2013). However, neither study examined the effects of comfort-drawing for *distressing* experiences – as examined in the present study. Since distress is a known memory inhibitor (Malta et al., 2020) we predicted that drawing something *comforting* (i.e., that *reduces* distress) during interviews would increase the total amount of information (word count) relative to those who talk-only. In addition, we predicted that the types of information reported would be more forensically relevant (e.g., objects, people, locations) compared to talk-only, replicating effects seen in other studies involving distressing events (e.g., Katz & Hershkowitz, 2010; Salmon et al., 2003; Wesson & Salmon, 2001). In line with Poole & Dickinson’s prediction that comfort-drawing may create a more supportive interview environment, we also predicted that comfort-drawing would enhance subjective wellbeing measures post‑interview, such as feelings of support and interview enjoyment, relative to event-drawing or talk-only. Analyses of voluntary memory were hypothesis-driven, whereas our examination of the effects of drawing on intrusion development was exploratory, and no specific predictions were made given the limited prior research examining the effects of drawing on memory intrusions. The project was preregistered on the Open Science Framework (https://osf.io/thkgm/overview) and approved by the Flinders University Human Research Ethics Committee (Project 2815).

## Method

### Participants

An a priori power analysis conducted in G*Power (Faul et al., 2007) using a fixed-effects one-way ANOVA indicated that 64 participants were required to detect a large effect (Cohen’s *f* = .40), with α = .05, 80% power, and three groups. The sample consisted of 59 students (20 event-drawing [14 female, 6 male], 19 comfort-drawing [12 female, 7 male], 20 talk-only control [12 female, 8 male]; *M* = 23.20, *SD* = 8.26) from Flinders University who each received course credit for their undergraduate psychology studies or a $15 AUD payment. Interviews were conducted either in person or via Microsoft Teams, with identical procedures across formats; participants completed the same Qualtrics questionnaires and interview tasks, differing only in whether these activities occurred in a physical laboratory setting or a virtual meeting environment.

#### Recruitment

Participants were recruited between March and May 2023 and between May and August 2024 through the university’s SONA research participation system, an online participant pool used by the School of Psychology. Eligible first-year psychology students (i.e., those who had experienced a negative/stressful event) could view study information, register for available studies and receive course credit for participation. Participation was voluntary, and alternative options for obtaining course credit were available.

##### Blinding

Researchers responsible for administering the intervention and conducting participant interviews were not blinded to condition allocation because delivery of the intervention required knowledge of participants’ assigned condition. However, researchers responsible for coding interview transcripts and scoring memory outcomes were blinded to participants’ experimental condition.

#### Measures

##### The Impact of Events Scale-Revised Questionnaire (IES-R)

The IES-R (Weiss & Marmar, 1997) contains 22 questions about how difficult things have been for the participant over the past 7 days in respect to a stressful life event and how bothered and/or distressed they have been by these difficulties. Participants rate each item using a 5-point scale (0 = *not at all*, 4 = *extremely*). Eight items correspond to symptoms of intrusions, 8 items correspond to symptoms of avoidance, and the remaining 8 items correspond to symptoms of hyperarousal. The mean rating for each subscale score can be interpreted according to symptoms of post-traumatic stress (PTS), where 0 = no symptoms, 1 = few symptoms, 2 = moderate symptoms, 3 = a high level of symptoms, 4 = an *extremely* high level of symptoms.

##### The Depression, Anxiety and Stress Scale (DASS-21)

The DASS-21 (Lovibond et al., 1995) was used to measure aspects of participants’ mental health. The DASS-21 is a structured self-report questionnaire consisting of a total 21 items, with 7 items per subscale: depression, anxiety and stress. Participants were asked to complete every item on the 4-point scale (0 = *did not apply to me at all*, 3 = *applied to me very much*). Sum scores were computed by adding up the scores on all the items and multiplying by a factor of 2. Total sum scores range from 0 to 120 and subscale scores range from 0 to 42.

### Conscious recall

#### Word count

The amount of information reported was calculated using the total word count from the transcribed audio interview recordings.

#### Statement categories

Interviews were transcribed by two independent research assistants who were blind to the interview conditions. Verbal interview transcripts were then coded for statements pertaining to each of the following categories: Objects, people, actions, locations, times, or affective statements (as per Gross & Hayne, 1998; Katz & Hershkowitz, 2010; Salmon et al., 2003; Wesson & Salmon, 2001). A score of 1 was given for each statement relating to a particular category (e.g., *“I was at the park”* = 1 point for location; *“I was at the park with my mother” =* 2 points, 1 pt for location and 1 pt for people).

### Intrusion measures

#### Intrusion frequency

In the intrusion task, participants were asked to press the “X” on the keyboard each time they experienced an intrusion while spending 5 min reading a 1,002‑word article about bread (as per Baird et al., 2013; M. K. Takarangi et al., 2014; M. K. T. Takarangi et al., 2017). The total number of “X” key presses was recorded using JavaScript in the Qualtrics survey software. The total number of key presses was used as the measure of intrusion frequency.

#### Intrusion qualities

Participants were also asked to rate overall qualities of their intrusions (i.e., their average experience of all intrusions) on a 7‑point scale (0 = *not at all*, 7 = *extremely*). Single item questions corresponded to qualities of distress, intensity, positive/negative emotion and vividness. Participants were also asked to rate 8 statements on a 7‑point scale (0 = *not at all*, 7 = *completely*) on how much they agreed with statements pertaining to attempts at thought suppression, such as “*I deliberately tried to bring images to mind*”, “*the images came to mind effortlessly*” (as per M. K. Takarangi et al., 2014).

### Additional interview measures

Pre- and post‑interview distress and task difficulty were measured on a sliding scale (0 = n*ot at all*, 100 = *extremely*). For the drawing conditions, task enjoyment and interviewer support were measured on a 7‑point scale (0 = *not at all*, 7 = *extremely*). Interview duration was recorded in minutes. Interview interruptions were coded by the research assistants who were blind to interview conditions. All transcripts were checked for any words spoken by the interviewer. Such occurrences were recorded as *interruptions* and included as covariates in all subsequent analyses. For example, the interviewer interjecting with *“ah, I see … ”* would equal a 3-word interruptions. In addition, any “off topic” words included by participants were also included as interruptions, for example, “ah” and “um”. Finally, all transcripts were reviewed and categorised into the types of experience shared (e.g., car accident, interpersonal conflict, medical emergency) by the first and third authors. Once transcripts were assigned a category, they were summed and the proportion of the sample that reported each experience type was recorded.

### Procedure

The study was conducted in a lab setting (*n* = 52) or online on a Microsoft Teams meeting (*n* = 7). Note that the distribution across conditions was similar for the online Teams participants (event-drawing, *n* = 2, comfort-drawing, *n* = 3, talk-only, *n* = 2). After providing consent, participants answered demographic questions (i.e., age, gender) and then completed the IES-R (Weiss & Marmar, 1997) and the DASS-21 (Lovibond et al., 1995) on Qualtrics. After completing the questionnaires, participants were asked to share one previous negative and/or stressful life experience that they felt comfortable sharing with the researcher. Specifically, participants were advised to share “a subjectively stressful experience from your past that you are willing to share with the researcher”. In the drawing conditions the additional instruction was provided, “I would also like you to draw *the experience/flowers of your choosing* using paper and pen/pencil. Please know that you are not being judged on the quality of your drawings, nor am I interpreting or assigning any ‘meaning’ to your drawings”. See Figure 1 for example drawings. They were reminded that the interview would be audio recorded for later transcription and coding, and that they were free to request the interview be stopped at any time without consequence. Participants were told that they had up to 30 minutes to provide as much detail as possible about their experience with the researcher. Prior to the interview commencing, participants were asked to indicate their feelings of pre-interview distress. Participants were then randomly allocated (on Qualtrics) to one of three conditions: 1) an *event-drawing* condition, in which participants were asked to draw their chosen experience while talking; 2) a *comfort-drawing* condition in which participants were asked to draw flowers while talking about their experience; or 3) a talk-only control condition in which they were asked to only verbally share their experience. Participants in the drawing conditions were asked to use a black pen and a sheet of white paper. Participants were informed that the researcher was not interested in the quality of their drawings, nor were they judging or making any inferences about the content/meaning of their drawings. Researchers provided minimal encouragers during the interview (e.g., *“ah”, “mmm”*) and refrained from asking follow-up questions.

**Figure 1.**
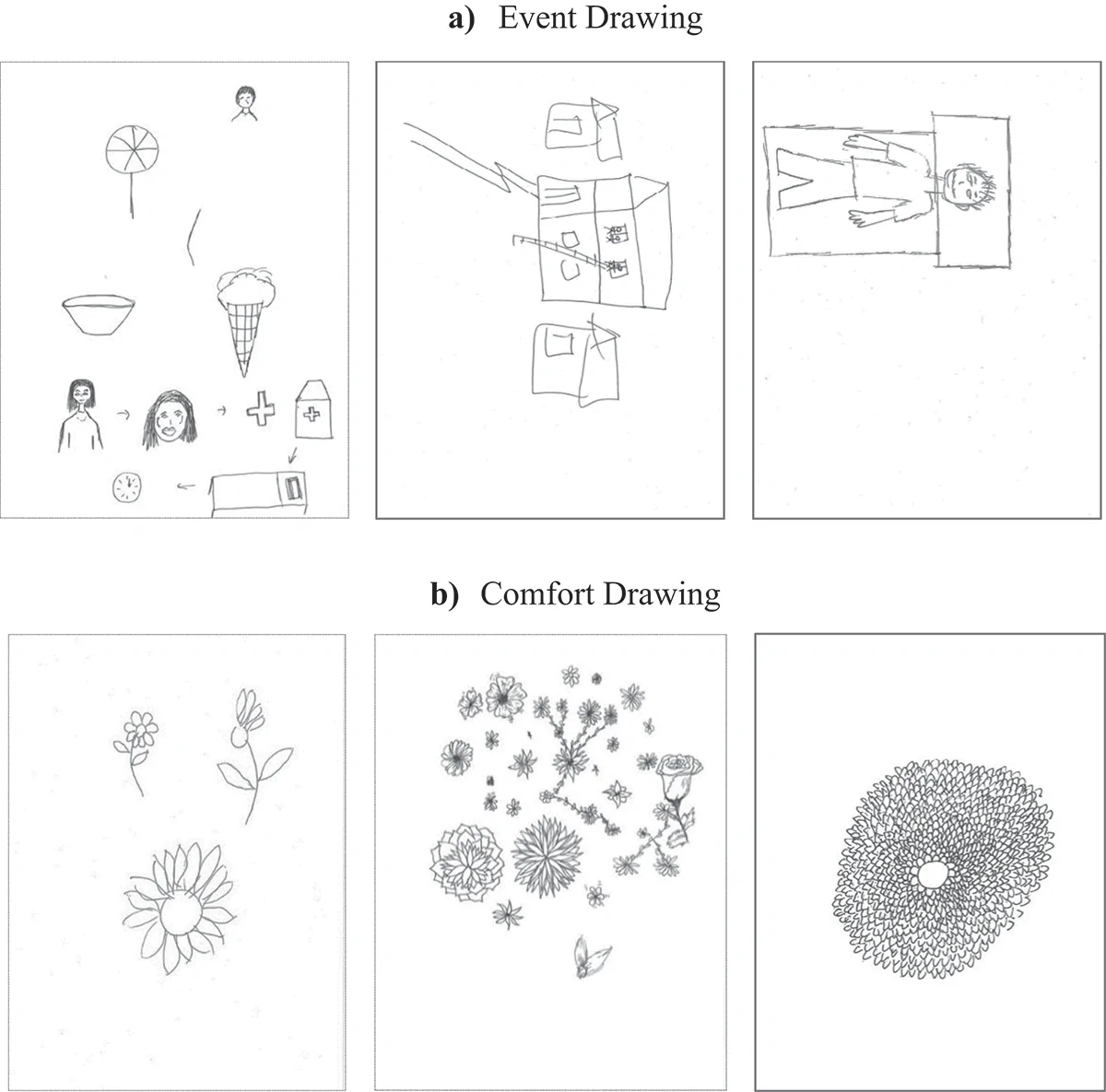
Drawing examples.

After participants had shared their experience, their drawings were collected by the researcher or uploaded online by the participant. They were then again asked to indicate their current feelings of distress (post-interview distress measure) and to rate how difficult they found sharing their experience on a sliding scale (0 = *not at all difficult*, 100 = *extremely difficult*). They were then asked to rate “*how enjoyable*” they found the drawing task and “*how supported*” they felt by the researcher when drawing, on a 7-point scale (0 = *not at all*, 7 = *extremely*). If they were allocated to the talk-only condition, they were asked to select NA = *not applicable*. Participants then received instructions on how to complete the intrusions task. In the intrusions task, participants read a 1002-word neutral article about bread (see M. K. Takarangi et al., 2014) and pressed the “X” on the keyboard each time they experienced an intrusion of the *event* they discussed while reading the article. Intrusions were defined as “involuntary recollections of experienced events, such as spontaneous images, thoughts, or a combination of both”. After reading the article, participants were asked to complete the intrusion qualities questions. At the conclusion of the study, all participants were verbally debriefed and received a debriefing email which included information about the study’s aims, purpose, justification and contact information for additional support services.

## Results

For completeness, all descriptive and inferential statistics are presented herein. Results are presented and discussed in terms of trends, with a specific focus on effect sizes for intrusion measures. The effect size benchmarks used for subsequent analyses were Cohen’s *d*, 0.2 = small, 0.5 = medium, 0.8 = large (Cohen, 1988) and eta-squared (*η*^*2*^), 0.01 = small, 0.06 = medium, 0.14 = large. No participants were excluded from the study; all available data were retained, with listwise deletion applied where data were missing for a particular analysis.

Table 1 presents the proportion of experience types shared by the sample. Participants were asked to choose events that were personally distressing, with remembered events ranging in severity from moving house through to witnessing a fatal car accident. Although we did not use a clinical PTSD sample in the present study, 45.27% of memories recalled were of experiences that would meet the requirements for Criterion A of a PTSD diagnosis (APA, 2022; see Table 1). Most participants shared an interpersonal conflict, such as an argument with a family member as their distressing experience during the interview. More participants shared a second-hand experience of having witnessed a medical emergency (e.g., a cardiac arrest or stroke) than a firsthand first-hand experience. However, more participants shared a first-hand experience of a mental health crisis than second-hand experiences (e.g., suicidal ideation disclosure). More participants shared a first-hand rather than second-hand car accident experience during the interview. Finally, participants also discussed first-hand experiences of grief and loss, as well as experiences of physical assault.

**Table 1.** Examples and proportions of distressing experiences shared during interview.

Experience Type	Percentage	Examples
Interpersonal Conflict	35.59%	Disagreement with close friend; argument with child; relationship break-up; other family conflict; conflict with work colleague
Car Accident Firsthand experience	8.47%	Motor vehicle accident
Car Accident Secondhand experience	3.38%	Witness to motor vehicle accident
Grief and Loss	6.77%	Death of a family member; death of a friend; death of pet
Medical Emergency Firsthand experience	13.55%	Hospitalisation due to unexpected injury; life threatening allergic reaction; substance induced psychosis
Medical Emergency Secondhand experience	8.47%	Witness to a cardiac arrest or stroke; witness to life threatening allergic reaction
Mental Health Episode Firsthand experience	11.86%	Moving house; academic pressure; substance induced psychosis
Mental Health Episode Secondhand experience	6.77%	Listening to sexual assault disclosure; listening to suicidal ideation disclosure; witness to substance induced psychosis
Physical Assault Firsthand experience	5.08%	Physically assaulted

The means and standard deviations for the DASS-21 and IES-R scores are presented in Table 2. There were no significant differences between groups on any of the DASS-21 scales or on any of the IES-R scales. Nonetheless, the comfort-drawing group had higher stress and intrusion scores than the other two conditions prior to the interview taking place, and as such, the DASS-21 and IES-R scores were entered as covariates in all subsequent analyses.

**Table 2.** One-way ANOVA of DASS-21 and IES-R subscale scores by task condition.

Measures	Event drawing (*n* = 20)	Comfort drawing (*n* = 19)	Talk-only (*n* = 20)		
	*M (SD)*	*M (SD)*	*M (SD)*	*F*(2, 56)	*p*
**DASS-21**					
Depression	4.75 (3.97)	6.11 (5.51)	4.20 (3.37)	0.98	.380
Anxiety	4.30 (3.48)	5.11 (3.67)	4.90 (3.88)	0.26	.776
Stress	7.15 (4.25)	8.26 (4.65)	7.50 (4.14)	0.33	.718
**IES-R**					
Intrusions	8.10 (6.66)	10.05 (6.64)	9.55 (7.22)	0.43	.652
Avoidance	10.50 (7.17)	8.26 (5.14)	8.95 (6.13)	0.67	.518
Hyperarousal	4.00 (4.65)	4.68 (4.07)	5.70 (4.68)	0.73	.487

### Conscious recall

Figure 2 illustrates group means for the total word count with 95% confidence intervals. Interrater reliability was acceptable (α = .89; see as per Cronbach’s alpha analysis for all categories coded (objects, people, actions, locations, times, or affective statements). Word count and voluntary memory statement categories were analysed using an ANCOVA. Covariates in the model for each analysis were DASS-21 (by subscale), IES-R total scores and interview interruptions (see Table 2).

**Figure 2.**
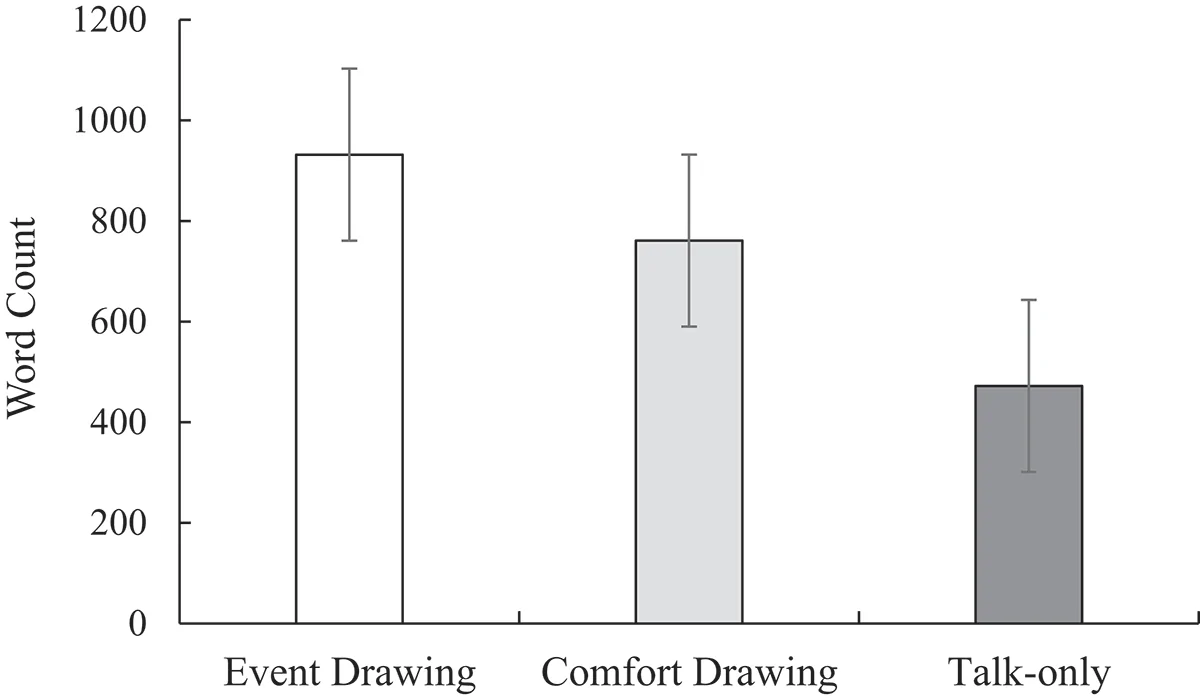
Effects of interview condition on conscious recall.

*Note*. Error bars represent the 95% CI, unadjusted for covariates.

#### Word count

Figure 2 illustrates word count scores by interview condition (event-drawing, comfort-drawing, talk-only control). The ANCOVA demonstrated a significant main effect of interview task on word count, *F*(2, 51) = 3.85, *p* = .028, *n*^*2*^ = 0.11. Post-hoc comparisons revealed that those in the event-drawing condition reported more information than the talk-only group, *95% CI* [−816.64, −125.15], *p* = .009. This difference remained significant when we applied a Bonferroni correction (*p* = .026) and was a large effect, *d* = 0.88, *95% CI* (−0.36, 0.97). There were no significant differences between the two drawing conditions *(p* = .636) or between the comfort-drawing condition and the talk-only control, *p = *.199.

#### Statement categories

Table 3 presents the results for all statement categories by task condition. As can be seen in Table 3, the only statement category that differed across interview conditions was *locations* (*p* = .044). Post-hoc comparisons revealed that the event-drawing condition reported significantly more locations during the interview than the comfort-drawing condition (*p* = .031, *d* = 0.73) and the talk-only condition (*p* = .031, *d* = 0.71), but these differences were not significant when we applied Bonferroni corrections (*p* = .092 for both comparisons).

**Table 3.** ANCOVA of voluntary memory statement categories by task condition.

Statement Category	Event drawing (*n* = 20)	Comfort drawing (*n* = 19)	Talk-only (*n* = 20)			
	*M (SD)*	*M (SD)*	*M (SD)*	*F*(2, 51)	*p*	*η*^*2*^
Objects	8.90 (12.37)	5.11 (10.10)	2.80 (4.34)	2.60	.084	.08
People	12.20 (19.84)	9.05 (8.96)	5.70 (6.00)	1.58	.216	.05
Actions	10.95 (10.88)	10.53 (8.65)	9.50 (9.71)	0.04	.964	.00
Locations	8.10 (11.08)	3.68 (4.08)	3.30 (5.34)	3.32	.044*	.10
Times	5.25 (5.43)	3.58 (3.67)	3.05 (3.10)	1.82	.172	.06
Affective	4.85 (4.58)	4.21 (5.01)	3.05 (2.76)	0.83	.442	.03

Note: Covariates in this analysis were DASS-21 scores (by subscale), IES-R total scores and interview interruptions.

### Intrusions

#### Intrusion frequency

Figure 3 shows group means with 95% confidence intervals for the total number of intrusions reported per task condition. There were no significant differences between interview conditions for intrusion frequency, *F*(2, 51) = 0.42, *p* = .659, *n*^*2*^ = .01.

**Figure 3.**
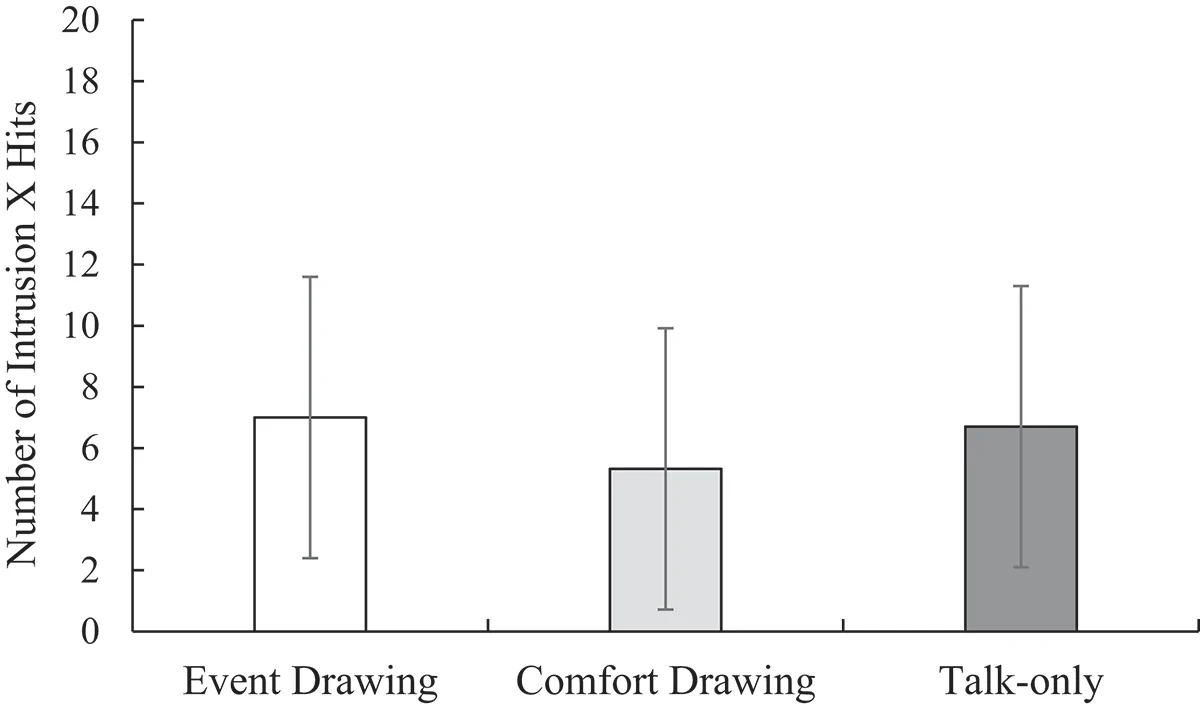
Effects of interview condition on intrusion measures.

Note: Error bars represent 95% CI, unadjusted for covariates.

#### Intrusion qualities

Table 4 illustrates the means and standard deviations of the intrusion qualities: distress, positive/negative emotion and vividness. Intrusion qualities (distress, positive/negative emotion, vividness) did not significantly differ across interview conditions.

**Table 4.** ANCOVA of involuntary memory intrusion qualities by task condition.

Intrusion Qualities	Event drawing (*n* = 20)	Comfort drawing (*n* = 19)	Talk-only (*n* = 20)			
	*M (SD)*	*M (SD)*	*M (SD)*	*F*(2, 51)	*p*	*η*^*2*^
Distress	2.75 (1.89)	3.42 (2.70)	2.63 (1.61)	0.20	.822	.01
Positive/Negative Emotion	3.05 (1.36)	2.68 (1.49)	3.05 (1.96)	0.75	.479	.02
Vividness	3.35 (1.35)	3.00 (1.53)	3.30 (1.59)	0.62	.541	.02

Note: Covariates in this analysis were DASS-21 scores (by subscale), IES-R total scores and interview interruptions.

### Additional interview measures

Table 5 illustrates the means and standard deviations of the additional interview measures for the pre- and post‑interview distress, task difficulty, interview duration and interruptions measures. There were no meaningful differences on any measure, though directionally, event-drawing interviews were longer than both comfort and talk-only interviews.

**Table 5.** One-way ANOVA of pre‑ and post-intervention distress, task difficulty, interview duration and interruptions by task condition.

Measures	Event drawing (*n* = 20)	Comfort drawing (*n* = 19)	Talk-only (*n* = 20)			
	*M (SD)*	*M (SD)*	*M (SD)*	*F*(2, 56)	*p*	*η*^*2*^
Pre-intervention Distress	34.45 (25.70)	23.11 (19.89)	22.20 (25.21)	1.63	.204	.06
Post-intervention Distress	38.95 (31.36)	33.68 (23.87)	33.65 (22.39)	0.27	.765	.01
Task Difficulty	38.95 (30.82)	36.68 (30.75)	41.05 (25.95)	0.11	.897	.00
Interview Duration	6.69 (5.77)	4.98 (5.15)	3.41 (3.04)	2.35	.105	.08
Interview Interruptions	13.20 (17.53)	15.00 (17.56)	10.95 (15.35)	0.28	.754	.01

## Discussion

Our study was the first to explore the effects of event versus comfort-drawing during an interview on both conscious recall and intrusions for an autobiographical memory of a distressing experience. In line with previous studies (e.g., Gross & Hayne, 1998; Katz & Hershkowitz, 2010; Salmon et al., 2003; Wesson & Salmon, 2001), drawing a distressing personal event while discussing it (event-drawing) increased the amount of information reported during the interview compared to talking only. In addition, we found support for the idea that event-drawing during an interview may facilitate the disclosure of more forensically relevant information. Specifically, providing participants with the opportunity to draw while discussing their distressing experiences resulted in recall of more information about locations – a finding consistent with Katz and Hershkowitz (2010). Given that reports in the event-drawing condition included strong visual references (objects, people, locations) relative to less-visual information (e.g., times, actions, affective statements), event-drawing may have enhanced memory by facilitating mental imagery – a known memory enhancer (C. J. Dando et al., 2020). Nonetheless, findings must be interpreted with caution given the small sample size in the present study.

Our findings that those in the event-drawing condition reported more information than the talk-only group also contradict previous literature about divided attention (e.g., Craik et al., 2018; Naveh-Benjamin & Brubaker, 2019). For example, research often demonstrates that completing two tasks simultaneously (dividing attention) can impair rather than benefit memory (Craik et al., 2018; Naveh-Benjamin & Brubaker, 2019). Instead, our data suggest that drawing while talking (about a negative experience) simultaneously in an interview does not incur a cost. Drawing the same event being discussed may be less cognitively demanding than tasks used in the divided attention literature (e.g., continuous choice reaction time tasks; Naveh-Benjamin & Brubaker, 2019). Drawing a related image is also likely less cognitively demanding than drawing an unrelated image (i.e., comfort-drawing) to an event being discussed, explaining the memory benefit of event-drawing over comfort-drawing.

Comfort-drawing had little effect on the *amount* or *type* of information reported, a finding consistent with Poole and Dickinson (2013) and MacLeod et al. (2016). However, our participants described unique autobiographical experiences, and as such, we were not able to gauge the *accuracy* of the information they reported. Thus, it remains possible that comfort-drawing had unseen drawbacks, such as increasing the number of incorrect statements reported during the interview (as per MacLeod et al., 2016). In addition, there was variability in the types of experiences discussed during interviews, including within each category (e.g., death of a family member, death of a family pet; see Table 5). Future studies could therefore better control for experience type and gauge the accuracy of autobiographical event recall by using a distressing staged event, such as a crime (e.g., Ihlebæk et al., 2003).

We also observed trends consistent with Wesson and Salmon (2001) such that drawing (whether event focused or comfort) increased the duration of the interview, which may be beneficial under some circumstances (e.g., keeping the interviewee engaged while waiting on further resources and/or experts to arrive). Yet, we did not have interviewers ask follow-up questions, akin to a real-world setting. Rather, our interviewers were instructed to mostly listen to the experiences being shared to provide experimental control, but this reduced our study’s ecological validity. For example, it may be possible that any interview questions that arise from visual cues evoked during event-drawing (e.g., what a suspect was wearing) also facilitate memory recall to a greater extent than the visual cues, if any, that arise from visually depicting a pre-determined image (e.g., flowers). To address these issues, future studies may benefit from implementing a standardised interview questionnaire and protocol (e.g., Cognitive Interview; Matsuo & Miura, 2016), while also measuring interviewer interruptions and controlling for them statistically. Implementing a standardised questionnaire would help to ascertain whether interview duration increases as a function of drawing or as a function of the questions asked of interviewees.

We did not find evidence that comfort-drawing reduces intrusions as per other non-event focused visual-motor tasks (for review, see Brewin, 2014). Indeed, no differences were found between event-drawing, comfort-drawing, or talking only during the interview on intrusion measures (frequency, distress, vividness, positive/negative emotion). However, directional trends were observed that may warrant further consideration; participants in the comfort-drawing interview condition reported numerically fewer intrusions post-interview than the event-drawing or talk-only conditions – a finding consistent with previous intrusion intervention studies (e.g., James et al., 2015). Thus, even if comfort-drawing does not enhance *conscious recall* relative to event-drawing, it may reduce immediate unwanted *intrusions* after forensic interviews. This possibility warrants future investigation, and future studies may also wish to assess intrusion reduction over a longer period (e.g., 1-week; Wessel et al., 2025).

We acknowledge that our examination of intrusion measures was exploratory, and the clinical utility of our findings remains to be seen. Additionally, as the a priori power analysis was based on voluntary memory recall, the study may have been underpowered to detect meaningful effects on intrusions and accordingly, these exploratory findings should be interpreted with caution. Additional studies will be needed to fully understand the effects of drawing on intrusions and on conscious recall, for that matter. For example, future research could also use sentiment analysis techniques to determine whether the drawing type in interviews affects factors such as the emotional valence or coherence of interview content.

The present findings should also be interpreted within the specific context of the drawing tasks examined here. Arts therapy encompasses a broad range of creative modalities, therapeutic processes and treatment goals that extend beyond event-focused or comfort-drawing. Consequently, the present findings are best viewed as informing the use of *drawing*-based interventions within arts therapy (as per Maddox et al., 2024b). Future research should examine whether similar effects emerge across other arts-based modalities and therapeutic contexts.

## Conclusion

Our study investigated the relative effects of event-drawing and comfort-drawing during an interview on autobiographical memory, relative to talking only. Our findings indicate that event-drawing may be an effective intervention for increasing autobiographical memory and forensically relevant information (e.g., locations, objects, people) in an interview about a subjectively distressing experience. Although we found no solid evidence that comfort-drawing enhances event recall or reduces unwanted intrusions, future studies should further explore these possibilities. Regardless of the outcomes, such research has important implications for professional practices that use drawing with vulnerable populations following distressing experiences, such as in forensic, clinical and therapeutic settings.

## Open scholarship

This article has earned the Center for Open Science badges for Open Materials and Preregistered. The materials are openly accessible at https://osf.io/thkgm/overview
